# Re-evaluating Fasting Guidelines for Invasive Cardiac Procedures: A Systematic Review and Meta-analysis of Randomized Clinical Trials

**DOI:** 10.1016/j.jscai.2025.103819

**Published:** 2025-09-16

**Authors:** Sebhat Erqou, Michael Kwok, Yongdeok Shin, Mohammed Motaweih, Eric Heinz, Aaron Schatz, Anna Tomdio, Luis A. Guzman

**Affiliations:** a Department of Medicine, Mary Washington Hospital, Fredericksburg, Virginia; b Department of Medicine, Research Services, Providence VA Medical Center, Providence, Rhode Island; c Department of Medicine, The Warren Alpert Medical School of Brown University, Providence, Rhode Island; d Department of Medicine, Washington University in St Louis, St Louis, Missouri; e Division of Anesthesiology, Mary Washington Hospital, Fredericksburg, Virginia

**Keywords:** cardiac procedures, fasting, meta-analysis, randomized controlled trials

## Abstract

**Background:**

Although questioned for more than a decade, fasting prior to invasive cardiac procedures remains the standard of care, due to lack of sufficient randomized data. We sought to synthesize data from existing randomized clinical trials (RCTs) comparing fasting vs nonfasting prior to cardiac procedures.

**Methods:**

We performed a systematic literature search for RCTs comparing fasting vs nonfasting prior to invasive cardiac procedures with moderate sedation. Data were pooled using a random-effects model meta-analysis. We report standardized mean differences (SMDs) or odds ratios (ORs) and 95% CIs.

**Results:**

Overall, 8 RCTs comprising of 3451 participants were included. The average fasting time was 845 minutes in the fasting group and 196 minutes in the nonfasting group. The pooled patient satisfaction score across 6 studies was SMD 0.78 (95% CI, 0.25-1.31) favoring the nonfasting arm. Hypotension (4 studies; OR, 1.6; 95% CI, 1.2-2.3) and hunger (3 studies; OR, 2.7; 95% CI, 1.8-3.7) were significantly higher in the fasting arm. There was a trend toward lower risk of contrast-induced nephropathy (OR, 0.7; 95% CI, 0.4-1.0) in the fasting arm across 4 studies with available data, but it did not reach statistical significance (*P* = .06). There was no difference in post- vs pre- procedure delta creatinine clearance (SMD, 0.07; 95% CI, −0.17 to 0.31) across 3 studies. There were also no significant differences in nausea/vomiting, hypoglycemia, pneumonia, and mortality.

**Conclusions:**

This synthesis of emergent clinical trial data suggests that nonfasting protocols for cardiac procedures are safe and associated with improved patient satisfaction. This study supports updating fasting guidelines for lower risk invasive cardiac procedures.

## Introduction

Although the need for fasting prior to invasive cardiac procedures has been questioned for over a decade,^1^ current guidelines for both cardiology and anesthesia recommend a minimum of 6 hours fasting for solids and 2 hours for clear liquids prior to these procedures.^2^^,^^3^ While the rationale for fasting has been to reduce the risk of vomiting and aspiration pneumonia that accompanies sedation,^2^^,^^4^ mounting observational data suggests that these are very rare complications after cardiac procedures in patients who do not receive general anesthetics.^5^, ^6^, ^7^ For example, a retrospective study of over 400 cases of emergency left heart catheterization for ST elevation myocardial infarction in which the patient population was not able to fast for the procedure identified only 1 case of likely aspiration pneumonia.^8^ In addition, several observational studies have reported that preprocedural fasting is associated with patient discomfort, dissatisfaction, and delays in treatment.^3^^,^^4^ The available evidence to date has been insufficient to change guidelines due to the lack of adequate randomized clinical trial (RCT) data. More recently, several clinical trials have been published^9^ expanding extant knowledge in the field; however, the data have not been adequately synthesized. We undertook a systematic review and meta-analysis of existing clinical trials comparing fasting vs nonfasting prior to invasive cardiac procedures.

## Materials and methods

### Search strategy

A comprehensive literature search was conducted to identify RCTs comparing nonfasting and fasting protocols in patients undergoing cardiac catheterization or related procedures. We searched the PubMed, Embase, and Cochrane Library databases using keywords related to "nonfasting," "fasting," "cardiac catheterization," "cardiac procedures," and "randomized controlled trials”. The electronic search was supplemented by manual reviews of references from included studies and relevant conference abstracts.

### Inclusion and exclusion criteria

To ensure the meta-analysis captured relevant and high-quality evidence, the following inclusion criteria were applied: (1) the study must be an RCT, (2) participants must be adults (≥18 years old) undergoing cardiac catheterization, cardiac implantable electronic device procedures, or other transcatheter cardiac procedures that do not require general anesthesia or monitored anesthesia care, and (3) the study must compare fasting with nonfasting protocols. The outcomes studied included procedural safety, adverse events, patient comfort, or clinical efficiency. Nonrandomized studies, observational studies, case reports, studies involving pediatric populations, and those lacking sufficient clinical outcome data or those not published in English were excluded.

### Data extraction

Two independent reviewers screened titles and abstracts of identified studies to determine eligibility. When available, full-text reviews were conducted for studies that met inclusion criteria. Information was collected using a standardized form by 3 authors (M.K., S.E., M.M.), which included details on study design, population characteristics, interventions, primary outcome definition, outcome measure, secondary outcomes, secondary outcome measures, and risk of bias. Discrepancies between reviewers were resolved through discussion or consultation with the senior author (L.G.).

### Quality assessment

The methodological quality of the included studies was assessed using the Cochrane Risk of Bias Tool. This tool evaluates several domains, including randomization, allocation concealment, blinding, and outcome reporting. Studies were categorized as having low, unclear, or high risk of bias in each domain.

### Statistical analysis

Summary study characteristics (eg, mean age, mean fasting time, sex, race) were pooled across studies by randomization groups using the sample size in each arm as analytical weights, providing estimates of weighted average means or percentages. The *P* values comparing summary study-level characteristics (means or prevalences pooled across the studies) between fasting and nonfasting arms were calculated from a linear regression model of each variable upon randomization status, weighted by the total sample size for each RCT (ie, fixed-effects meta-regression). Reported primary outcomes varied across studies and could not be uniformly pooled in meta-analysis and therefore are presented in tabular form. Patient satisfaction score was taken as the main outcome measure for the meta-analysis, as it was reported as the primary or secondary outcome for most studies. This was pooled across the studies with random-effects model meta-analysis using sample size, mean score, and SD score data in each randomization arm. The pooled effect measure is expressed as standardized mean difference (SMD) and 95% CI. For patient satisfaction outcome, studies generally coded higher scores to indicate less satisfaction, except for 1 study^10^ in which higher scores were coded to indicate greater satisfaction. For this study, the contrast for the satisfaction score was reversed prior to combining data in the meta-analysis, to be consistent with the remaining studies. The hospital length of stay outcome was pooled similarly. Secondary outcomes included hunger, nausea/vomiting, hypotension, hypoglycemia, contrast-induced nephropathy (CIN), pneumonia, and death. The number of events in each randomization arm were calculated from the percentage of events and the total sample size in the respective arm. These outcomes were pooled across the studies, using the number events in each arm and the total sample size in each arm, with a random-effects meta-analysis model. The pooled effect measure is expressed as odds ratio (OR) and 95% CI. For studies that reported pre- and post-procedural estimated glomerular filtration rate (eGFR), we calculated the change in eGFR for each study and then pooled it across the studies using random-effects model meta-analysis, with results expressed as SMD and 95% CI. We also combined the patient satisfaction score data using a fixed-effect meta-analysis model in sensitivity analyses.

We assessed heterogeneity between studies using Cochran’s Q statistic and the *I*^2^ statistic, which estimates the percentage of total variation across studies due to true between-study differences rather than chance, considering *I*^2^ cut-off values of 25%, 50%, and 75% to represent low, medium, and high heterogeneity, respectively.^11^^,^^12^ We did not explore heterogeneity further due to the limited numbers of studies.

For patient satisfaction score outcome, we visually inspected funnel plots to assess the risk of publication bias, which was also formally tested using the Egger test. In addition, we performed influence analysis to assess if the pooled patient satisfaction score was unduly affected by any single study. A 2-sided *P* value of < .05 was considered statistically significant. All analyses were performed using Stata version 15 (StataCorp LLC). We report this study according to Preferred Reporting Items for Systematic Reviews and Meta-Analyses (PRISMA) guidelines.^13^ The study did not require institutional review board approval as it is a meta-analysis of already published clinical trials.

## Results

The Medline search identified 8867 articles, of which 1347 were clinical trials. This was supplemented with a search of other electronic databases and a manual search as detailed above. Eight RCTs^10^^,^^14^, ^15^, ^16^, ^17^, ^18^, ^19^, ^20^, ^21^ with a total of 3451 participants were included in the meta-analysis. Two of the RCTs^16^^,^^17^^,^^19^ were published as abstracts for conference proceedings, with 1 study^16^^,^^17^ presented in 2 conferences at the interim and final analysis stages. One RCT^22^ that evaluated patients receiving transcatheter aortic valve replacement or atrial fibrillation ablation under general or monitored anesthesia care was excluded. The characteristics of individual studies are detailed in Table 1. Six studies^10^^,^^14^, ^15^, ^16^, ^17^^,^^19^ included coronary angiography and/or percutaneous coronary intervention (PCI) procedures, with 1 study^15^ also including right heart catheterization procedures. One study^21^ included coronary angiography, PCI, and cardioverter defibrillator implantation, while another study^20^ only assessed cardioverter defibrillator implantation and related procedures. Studies generally excluded patients with significant gastrointestinal pathologies, such as gastroparesis or total parenteral nutrition dependency, and those needing emergent catheterization.

**Table 1:** Summary of 8 randomized controlled trials fasting vs nonfasting prior to cardiac procedures.

Trial	Reference, year	Design	Location	Population	Procedure	Exclusion	No.		Outpatient (%)		Male (%)		Age (y)	
							F	NF	F	NF	F	NF	F	NF
TONIC	Boukantar et al,^14^ 2024	Single-blinded noninferiority RCT	France	Adults >18 y; Henri Mondor Hospital, Cardiology Dept	Coronary angio, PCI	Unstable, ACS, pregnant, lactating, simultaneous noncoronary procedures	360	365	69	65	76	75	67	68
CALORI	Mitchell et al,^15^ 2024	Single-center RCT	USA	Adults >18 y; Hospital in Virginia	Elective or urgent LHC, coronary angio, PCI, or RHC	Pregnancy, BMP >45, dementia, severe GERD, encephalopathy, chronic pain medications	94	104	NR	NR	63.8	65.4	61.5	60.5
CHOW NOW^a^	Mishra et al,^16^ 2020 Mishra et al,^17^ 2019	Single-center, single-blinded RCT	USA	Guthrie Hospital in Pennsylvania	Elective cardiac cath	NR	306	293	50	50	63	64	66.8	67.8
Woods C	Woods et al,^18^ 2024	Single-center, single-blinded RCT	USA	Adults >18 y; elective inpatient cath in-hospital in Indiana	Elective cath (LHC) with conscious sedation	Chronic nausea or vomiting, large hiatal hernia, enteral feeding, emergent cath	97	100	0	0	62	62	63	63
Li Y^a^	Li et al,^19^ 2017	Single-center RCT	Singapore	Elective or in-hospital cardiac cath	Elective or in-hospital cardiac cath	Emergent procedures, SpO_2_ <92%, dysphagia, Hx of CVA	266	249	10.2	61.4	79.7	73.9	62.5	62.3
Fast-CIED	Bode et al,^20^ 2022	Single-blinded RCT	Germany	Adults >18 y; elective CIED procedure; Leipzig Heart Center	New CIED implantation, battery exchange, lead revision	BMI >40, severe intra-abdominal tumors/ascites, bradycardia HR <30, unstable, TVP, intubated/deep sedation	101	100	0	0	66.3	67	72.5	71.6
CORO-NF	Tamborrino et al,^10^ 2024	Single-center RCT	Italy	Adults >18 y; Pisa University Hospital	Elective coronary angiography	Intubated, TPN, parental or NGT enteral nutrition	150	150	NR	NR	74.6	66.7	69	69
SCOFF	Ferreira et al,^21^ 2024	Multicenter, noninferiority RCT, blinded end point	Australia	Adults >18 y across 3 procedure sites in New South Wales	Coronary angiography, PCI, CIED	Requirement for GA, emergent procedures, structural interventions, planned atherectomy/lithotripsy, EP study, CRT	358	358	65.9	65.1	64.5	65.9	70	69
Pooled	—	—	—	—	—	—	1732	1719	44.7	52.5	69.8	68.5	67	67

ACS, acute coronary syndrome; angio, angiography; BMI, body mass index; cath, catheterization; CIED, cardiac implantable electrical defibrillator; CRT, cardiac resynchronization therapy; EP, electrophysiology; CVA, cerebrovascular accident; F, fasting; GA, general anesthesia; GERD, gastroesophageal reflux disease; HR, heart rate; Hx, history; LHC, left heart catheterization; NA, not applicable; NF, nonfasting; NGT, nasogastric tube; NR, not reported; PCI, percutaneous coronary intervention; RCT, randomized controlled trial; RHC, right heart catheterization; SpO_2_, peripheral capillary oxygen saturation; TPN, total parenteral nutrition; TVP, transvenous pacing.

a These studies were available as abstracts of scientific conference presentations. Data for CHOW NOW study obtained from 2 abstracts.

The summary characteristics pooled across the studies are shown in Supplemental Table S1. Overall,1732 participants (mean age 67 years, 70% men) were randomized to the fasting arm and 1719 (mean age 67 years, 69% men) to the nonfasting arm. Of the patients, 45% in the fasting arm and 53% in the nonfasting arm were outpatients. The average nothing-by-mouth time was 845 minutes (range, 757-970 minutes) in the fasting group and 196 minutes (range, 148-312 minutes) in the nonfasting group.

The primary outcome for each trial is shown in Table 2. The studies used 2 broad types of primary outcome measures: (1) patient satisfaction score or well-being score composed of some combination of hunger, thirst, headache, nausea, etc.; and (2) a composite clinical outcome comprising some combination of CIN, hypotension, hypoglycemia, aspiration pneumonia, etc. Overall, the nonfasting group reported a significantly higher satisfaction score than the fasting group, while the 2 groups were comparable in terms of the composite clinical outcomes (Table 2).

**Table 2:** Summary of primary to outcomes reported in 8 randomized controlled trials of fasting vs nonfasting prior to cardiac procedures.

Trial	Reference, year	Fast time (min)		Primary outcome			
		Fasting	Nonfasting	Definition	Fasting	Nonfasting	*P*
TONIC	Boukantar et al,^14^ 2024	900	180	Vasovagal symptoms, hypoglycemia, nausea/vomiting (up to 4 h post procedure)	9.90%	8.20%	ns
CALORI	Mitchell et al,^15^ 2024	970	148	Well-being score hunger/tiredness, intra- and postprocedural adverse events (emesis, aspiration, need for intubation)	6.0 ± 2.5	2.4 ± 2.4	<.001
CHOW NOW^a^	Mishra et al^16^, 2020 Mishra et al^17^, 2019	NR	NR	Composite of CIN, hypotension, aspiration pneumonia, nausea, vomiting, hypoglycemia, and hyperglycemia	9.80%	11.30%	.65
Woods et al	Woods et al,^18^ 2024	NR	NR	Satisfaction score	3.1 ± 1.5	1.3 ± 0.7	<.01
Li et al^a^	Li et al,^19^ 2017	NR	NR	NR	NR	NR	NR
Fast-CIED	Bode et al,^20^ 2022	758	312	Well-being score (hunger, tiredness, thirst, headache, nausea) + incidence of intraprocedural adverse events	16.5 ± 11.4	13.1 ± 9.6	.029
CORO-NF	Tamborrino et al,^10^ 2024	821	230	Satisfaction score + frequency of complications related to contrast media administration	4.2 ± 0.7	2.9 ± 1.2	<.001
SCOFF	Ferreira et al,^21^ 2024	792 (solid), 420 (liquid)	180 (solid), 144 (liquid)	Composite of aspiration pneumonia, hypotension, hyperglycemia, hypoglycemia	19.10%	12%	<.05

CIN, contrast-induced nephropathy; NR, not reported; ns, not significant.

a These studies were available as abstracts of scientific conference presentations. Data for CHOW NOW study obtained from 2 abstracts.

Central Illustration (panel A) shows patient satisfaction score, the main primary end point of the meta-analysis. The pooled patient satisfaction score across 6 studies was SMD 0.78 (95% CI, 0.25-1.31) for the fasting vs nonfasting arms, favoring the nonfasting group (higher value indicates less satisfaction; the definition of satisfaction score in each study is shown in Supplemental Table S2). There was no significant difference in terms of length of hospital stay duration between fasting and nonfasting groups (3 studies; SMD, 0.24; 95% CI, −0.45 to 0.93; Supplemental Figure S1). Central Illustration (panel B) displays the pooled analyses for individual clinical outcomes. Hypotension (4 studies; OR, 1.6; 95% CI, 1.2-2.3) and hunger (3 studies; OR, 2.7; 95% CI, 1.8-3.7) were significantly higher in the fasting arm than in the nonfasting arm. There was a trend toward lower risk of CIN (OR, 0.7; 95% CI, 0.4-1.0) in the fasting arm than in the nonfasting arm across 4 studies with available data, but it was not statistically significant (*P* = .06; Central Illustration panel B). Similarly, the pre vs post-procedural difference in eGFR (renal function) was similar between the fasting and nonfasting arms (SMD, 0.07; 95% CI, −0.17 to 0.31) across 3 studies (Central Illustration, panel C). Nausea/vomiting, hypoglycemia, pneumonia, and mortality did not differ significantly between the fasting and nonfasting groups. However, data for aspiration pneumonia and mortality outcomes were especially sparse. Sensitivity analysis yielded similar results on patient satisfaction score when data were pooled using fixed-effect mode meta-analysis (Supplemental Figure S2)

**Central Illustration:**
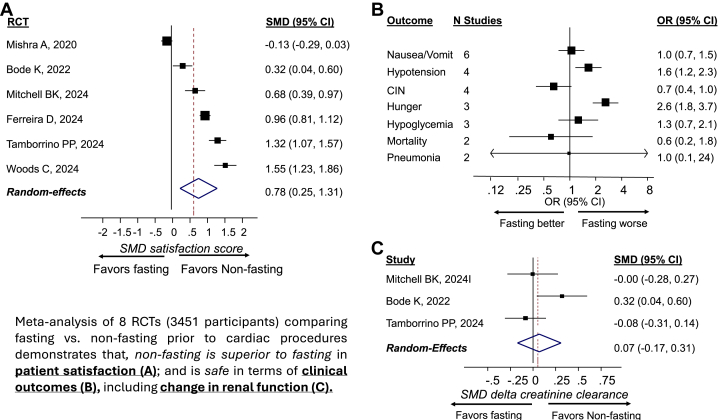
**Fasting vs nonfasting prior to cardiac procedure: meta-analysis.** (A) Patient satisfaction score in 6 RCTs comparing fasting vs nonfasting prior to cardiac procedures. (B) Seven clinical outcomes in 8 RCTs comparing fasting vs nonfasting prior to cardiac procedures (random-effects model meta-analysis). (C) Change in renal function (creatinine clearance) before vs after cardiac procedures across 3 RCTs comparing fasting and nonfasting groups. The study specific effect estimates were pooled using random-effects model meta-analysis. CIN, contrast-induced nephropathy; OR, odds ratio; RCT, randomized controlled trial; SMD, standardized mean difference.

There was significant heterogeneity across the studies for patient satisfaction score outcome (*I*^2^ = 97%, *P* < .001). There were too few studies to perform informative heterogeneity analysis. Visual inspection of the funnel plot and Egger test for 6 studies reporting patient satisfaction scores did not indicate significant publication bias (*P* = .565; Supplemental Figure S3). Influence analyses suggested no study had an undue influence on the pooled estimates for patient satisfaction score outcome (Supplemental Figure S4). Further sensitivity analysis of the pooled estimate for patient satisfaction score excluding the 2 studies that were only available as abstracts of conference proceedings^16^^,^^17^^,^^19^ yielded similar results (Supplemental Figure S5). The Cochrane Tool for Risk of Bias suggested that the included RCTs overall have low concern or some concern for bias (Supplemental Table S3).

## Discussion

In a meta-analysis of 8 RCTs involving 3451 participants comparing preprocedural fasting vs nonfasting before elective invasive cardiac procedures, we found that nonfasting was noninferior to fasting in terms of several clinical outcomes and was significantly superior to fasting in patient satisfaction. The fasting group was 2.7-fold more likely to report hunger, 1.6-fold more likely to experience hypotension, and more likely to score higher in patient dissatisfaction (SMD, 0.8). While the risk of CIN trended lower across a few studies (OR, 0.7), the results did not reach full statistical significance, and the difference between pre- and post-procedural eGFR was similar between the fasting and nonfasting arms (SMD, 0.07).

This meta-analysis provides an updated synthesis of the available clinical trial data comparing fasting vs nonfasting recommendations prior to cardiac procedures. The current study corroborates previous observational reports^6^, ^7^, ^8^ indicating that the incidence of aspiration pneumonia is extremely low in nonfasted states, such as with emergent cardiac procedures for which fasting is not feasible. Furthermore, the incidence of asphyxiation was previously found to be comparable between emergent (nonfasted) and nonemergent (fasted) PCI procedures.^6^ This study augments prior observational reports by providing randomized trial data on a broader range of outcomes, including patient satisfaction, hunger, nausea, vomiting, hypoglycemia, and CIN. The study confirmed the safety of nonfasted protocols in terms of feared procedural complications such as vomiting and aspiration pneumonia and their superiority in terms of hunger and patient satisfaction compared with fasting protocols. While our meta-analysis focused on cardiac procedures that did not require general or monitored anesthesia care, one recent RCT^22^ looking at transaortic valve replacement or atrial fibrillation ablation reported similar results. These findings lend further evidence for reconsideration of current fasting guidelines prior to elective and urgent invasive cardiac procedures.^4^^,^^5^ An interdisciplinary review by cardiology and anesthesiology societies is warranted.

Contrary to our expectation, we found a trend toward a lower risk of CIN in the fasting protocol arm across 4 studies with available data, but the association was not statistically significant. Further, this does not represent the totality of the available randomized evidence on fasting status and procedure-associated changes in renal function, since pooled data from 3 studies comparing pre- and post-procedural creatinine clearance between fasting and nonfasting arms did not show significant difference. Insufficient data prevented pooling ORs of CIN or SMD in creatinine clearance before and after procedures across all 6 studies.

Dehydration and hypovolemia, which are expected to occur with prolonged fasting, are associated with significantly increased risk of CIN.^23^ The trend toward lower risk of CIN in the fasting arm across the 4 studies^14^^,^^16^^,^^21^ in which these data were available may be a spurious finding, given the similar pre- and postprocedural change in creatinine clearance between fasting and nonfasting arms reported in the other 3 RCTs,^10^^,^^15^^,^^20^ as discussed above. Alternatively, there may actually be a lower risk of CIN in the fasting arm in the studies, perhaps due to treatment bias from unblinded hydration management (ie, providers giving more intravenous fluid to individuals assigned to the fasting arm). While 2 of the studies reporting CIN outcome^14^^,^^16^ were single-blinded, in the third study,^21^ the proceduralist was not blinded to the allocation arm. One of the 2 single-blinded studies^14^ reported that the treating physicians in the wards were not blinded to the allocation status, while further detail was not available for the other study,^16^ which was published as an abstract. Based on these data, it may be inferred that the treating practitioner could have administered more intravenous hydration to patients who were fasting. Additional individual level data on the amount of intravenous fluid administered during the periprocedural period would be required to confirm this hypothesis.

Our meta-analysis has limitations. This is a literature-based aggregate data meta-analysis. Individual level data were not available, which precluded precise calculations of some estimates or evaluation of the effect of clinically relevant subcategories (such as sex or age). Second, there was substantial heterogeneity across the studies that could not be explored due to the small number of studies (8 RCTs) that were available for inclusion, limiting our ability to conduct meta-regression analyses or important subgroup analyses. Third, the individual RCTs were small, and 2 of them were only published in abstract format, further limiting the available evidence to answer the question. Many of the outcomes studied were reported by only a few studies, except for satisfaction score, which was reported by 7 studies, hunger by 6 studies, and hypotension by 5 studies. Data were particularly sparse and underpowered for pneumonia and mortality outcomes. Fourth, we were not able to investigate the relationship between fasting and cardiac procedure-associated change in renal function because data on risk of CIN and change in creatinine clearance could not be combined across the 6 studies that provided this information. Instead, we had conflicting results from the 4 studies reporting on risk of CIN vs the 3 studies reporting on pre- and post-procedural creatinine clearance. Furthermore, the lack of individual level data prevented assessment of the impact of differential intravenous fluid administration on the findings. Finally, we were not able to address important cardiac procedure-related outcomes that may have been affected by fasting, such as procedural delays and cancellations, as these were generally not studied in the RCTs. Although we present limited data on hypoglycemia, the impact of fasting (vs nonfasting) on glycemic management in patients with diabetes was not adequately explored.

Our study has several strengths that merit consideration. First, we conducted a comprehensive meta-analysis of the available random evidence of clinical outcomes comparing fasting vs nonfasting prior to elective cardiac procedures. The included RCTs are considered to have low to moderate risk for bias. Meta-analysis of good quality RCTs is considered the highest in the hierarchy of clinical evidence. Second, there is a strong background of biological and observational data to support the conclusions of the study. Third, despite the presence of heterogeneity, pooled data from random-effects and fixed-effect models were comparable, supporting the conclusions of the study findings. Further, the statistical analysis showed no indication of publication bias.

### Clinical perspective

Even though questioned for more than a decade, fasting prior to invasive cardiac procedures remains the standard of care and is included in both anesthesia and cardiology guidelines. Clinical practitioners have long noted that fasting prior to cardiac procedures tend to be associated with patient hunger, inconvenience, and dissatisfaction, as well as safety concerns such as difficulty in managing glucose-lowering medications in patients with diabetes. Disruption of clinical workflow and patient and caregiver inconvenience due to delayed, rescheduled, or cancelled procedures because of inadequate fasting can cause challenges. Past nonrandomized studies have supported these clinical observations and have not found evidence of an association between cardiac procedures performed in nonfasted state and risk of aspiration or pneumonia. However, the lack of good quality randomized data has made changes in guidelines challenging until recently. In this meta-analysis, we provide a comprehensive synthesis of emergent clinical trial data on the topic comprising 8 RCTs, including 5 that were published in the past year. We found that nonfasting protocols for cardiac procedures are safe and associated with improved patient satisfaction. These findings indicate the need to reconsider traditional fasting guidelines in favor of allowing more liberal oral intake before invasive cardiac procedures.

## Conclusion

The totality of the available evidence from randomized clinical data suggests that nonfasting protocols for invasive cardiac procedures are safe and associated with improved patient satisfaction. *This study* *supports updating fasting guidelines for low-risk cardiac procedures to enhance patient-centered care without compromising safety.*
