# Supplementary material for: Re-evaluating Fasting Guidelines for Invasive Cardiac Procedures: A Systematic Review and Meta-analysis of Randomized Clinical Trials
Source: J Soc Cardiovasc Angiogr Interv. 2025 Sep 16;4(10Part A):103819. doi: 10.1016/j.jscai.2025.103819 (PMC12629744; doi:10.1016/j.jscai.2025.103819)
Supplement: Supplemental Material [file mmc1.docx]

Supplemental Appendix

**Re-evaluating Fasting Guidelines for Invasive Cardiac Procedures: A Systematic Review and Meta-analysis of Randomized Clinical Trials.**

Supplement Table 1. Summary of available study characteristics across 9 randomized controlled trials included in meta-analysis……………Pg 2

Supplement Table 2. Definition of satisfaction score in each of the 8 RCTs included in meta-analysis……………Pg 3

Supplement Table 3. Cochrane risk of bias assessment for 8 RCTs included in meta-analysis……………Pg 4

Supplement Figure 1: Hospital stay in 3 RCTs comparing fasting vs non-fasting protocols prior to elective cardiac procedures……………Pg 5

Supplement Figure 2. Seven clinical outcomes in 8 RCTs comparing fasting vs. non-fasting prior to cardiac procedures (pooled using fixed-effect meta-analysis model) ……………Pg 6

Supplement Figure 3. Funnel plot to assess publication bias across 6 studies included in meta-analysis of patient satisfaction score……………Pg 7

Supplement Figure 4. Influence analysis of 6 randomized trials included in meta-analysis of patient satisfaction score……………Pg 8

Supplement Figure 5. Sensitivity analysis for meta-analysis of patient satisfaction score, excluding two studies* that were reported as abstract only. ……………Pg 9

Supplement Table 1. Summary of available study characteristics across 9 randomized controlled trials included in meta-analysis.

| **Variable** | **Studies** | **Fasting** | | | | **Non-fasting** | | | | | **P-value** |
| --- | --- | --- | --- | --- | --- | --- | --- | --- | --- | --- | --- |
|  |  | **N** | **Pooled mean** | **Minimum** | **Maximum** | **N** | **Pooled mean** | **Minimum** | **Maximum** |  | |
| N | 8 | 1732 | 1732 | 94 | 360 | 1719 | 1719 | 100 | 365 |  | |
| Outpatient (%) | 6 | 1488 | 44.7 | 0 | 69 | 1465 | 52.5 | 0 | 65.1 | 0.62 | |
| Mean Fasting Time (minutes) | 5 | 1063 | 845.1 | 757 | 970 | 1077 | 196.1 | 148 | 312 | <0.001 | |
| Male (%) | 8 | 1732 | 69.8 | 62 | 79.7 | 1719 | 68.5 | 62 | 75 | 0.70 | |
| Mean Age (yrs) | 8 | 1732 | 66.9 | 61.5 | 72.5 | 1719 | 66.9 | 60.5 | 71.6 | 0.98 | |
| Black (%) | 2 | 191 | 19.3 | 2 | 37.2 | 204 | 25.0 | 2 | 47.1 | 0.86 | |
| White (%) | 2 | 191 | 78.1 | 59.6 | 96 | 204 | 72.0 | 49 | 96 | 0.86 | |
| Mean BMI (kg/2) | 4 | 859 | 29.8 | 27.9 | 30.9 | 855 | 29.9 | 28.4 | 30.9 | 0.89 | |
| Current smoker (%) | 3 | 1024 | 17.5 | 13.8 | 23 | 1016 | 19.7 | 17.1 | 23 | 0.55 | |
| DM (%) | 7 | 1635 | 34.9 | 27.1 | 50 | 1619 | 36.5 | 26.5 | 50.6 | 0.74 | |
| HTN (%) | 6 | 1541 | 72.6 | 68.8 | 79.6 | 1515 | 70.6 | 65.5 | 76 | 0.43 | |

Supplement Table 2. Definition of satisfaction score in each of the 8 RCTs included in meta-analysis.

| **Trial** | **Definition of Satisfaction Score** |
| --- | --- |
| **TONIC** | Satisfaction score included hunger(either very hungry/hungry, not hungry or completely full), thirst (very thirsty/thirsty, not thirsty, completely hydrated), periprocedural pain( self-assessed using visual analog scale from 1{ no pain at all} to 10{extreme pain}, and feeding preference( fasting or ad libitium feeding) , overall procedure satisfaction(very satisfied, satisfied, neutral or not satisfied) |
| **CALORI** | Pre-procedure discomfort score( sum of hunger and fatigue on NRS{0-5} the higher the value the less satisfaction) |
| **CHOW NOW** | Not available |
| **Wood et al** | Scored as 1=strongly agree, 5 =strongly disagree with the statement that they were satisfied with the diet |
| **Li et al** | Not reported |
| **Fast-CIED** | Well-being score items (tiredness, weakness, dizziness, anxiety, hunger, thirst, mouth dryness, headache, stomachache, other pains, and nausea. Quantified on NRC from 0 (an item not existent) to 10 (an item in the most severe form) |
| **CORO-NF** | Satisfaction score ranging from 1 (maximum complaint/no approval) to 5 (minimum or no complaint/full approval). |
| **SCOFF** | Fasting questionnaire: 6 questions about fasting symptoms(thirst, hunger, hoarseness, anxiety, nausea, weakness), with 5-point ordinal scale ranging from strongly disagree to strongly agree with higher scores corresponds to worse symptoms( lower level of satisfaction) |
| **Atkinson et al** | Patient-reported thirst, hunger, headache, nausea, lightheadedness, and anxiousness prior to surgery.0–10 along a visual analog scale with lower values indicating greater satisfaction. |

Supplement Table 3. Cochrane risk of bias assessment for 8 RCTs included in meta-analysis.

| **Trial** | **TONIC** | **CALORI** | **CHOW NOW** | **Woods et al** | **Li et al** | **Fast-CIED** | **CORO-NF** | **SCOFF** |
| --- | --- | --- | --- | --- | --- | --- | --- | --- |
| **Random allocation** | Y | Y | Y | Y | Y | Y | N | Y |
| **Concealed allocation** | Y | Y | PY | PY | U | Y | N | PY |
| **Baseline difference?** | N | N | N | N | N | N | PN | N |
| **Risk-of-bias judgement** | Low | Low | Low | Low | Low | Low | Low | Low |
| **Participants Aware?** | Y | Y | Y | Y | Y | Y | Y | Y |
| **Interventionalist Aware?** | N | Y | PN | PY | PY | N | Y | Y |
| **Assignment analyses?** | N | N | N | N | N | N | N | N |
| **Risk-of-bias judgement** | Low | Some | Some | Some | Some | Low | Some | Some |
| **Data for all or nearly all?** | Y | Y | Y | Y | Y | Y | Y | Y |
| **Informative missingness?** | PN | PN | PN | PN | PN | PN | PN | PN |
| **Risk-of-bias judgement** | Low | Low | Low | Low | Low | Low | Low | Low |
| **Inappropriate measure?** | N | N | N | N | N | N | N | N |
| **Differential ascertainment** | PN | PN | PN | PN | PN | PN | PN | PN |
| **Assessor aware of assignment?** | PN | PY | PN | PY | PY | PN | PY | PN |
| **Risk-of-bias judgement** | Low | Low | Low | Low | Low | Low | Low | Low |
| **Pre-specified analysis?** | PY | PY | PY | PY | PY | PY | PY | PY |
| **Multiple eligible outcomes?** | Y | Y | Y | Y | Y | Y | Y | Y |
| **Multiple eligible analyses?** | Y | Y | Y | Y | Y | Y | Y | Y |
| **Risk-of-bias judgement** | Some | Some | Some | Some | Some | Some | Some | Some |
| **Overall Risk-of-Bias Judgement** | Low | Some | Low | Some | Some | Low | Low | Some |

Supplement Figure 1: Hospital stay in 3 RCTs comparing fasting vs non-fasting protocols prior to elective cardiac procedures.


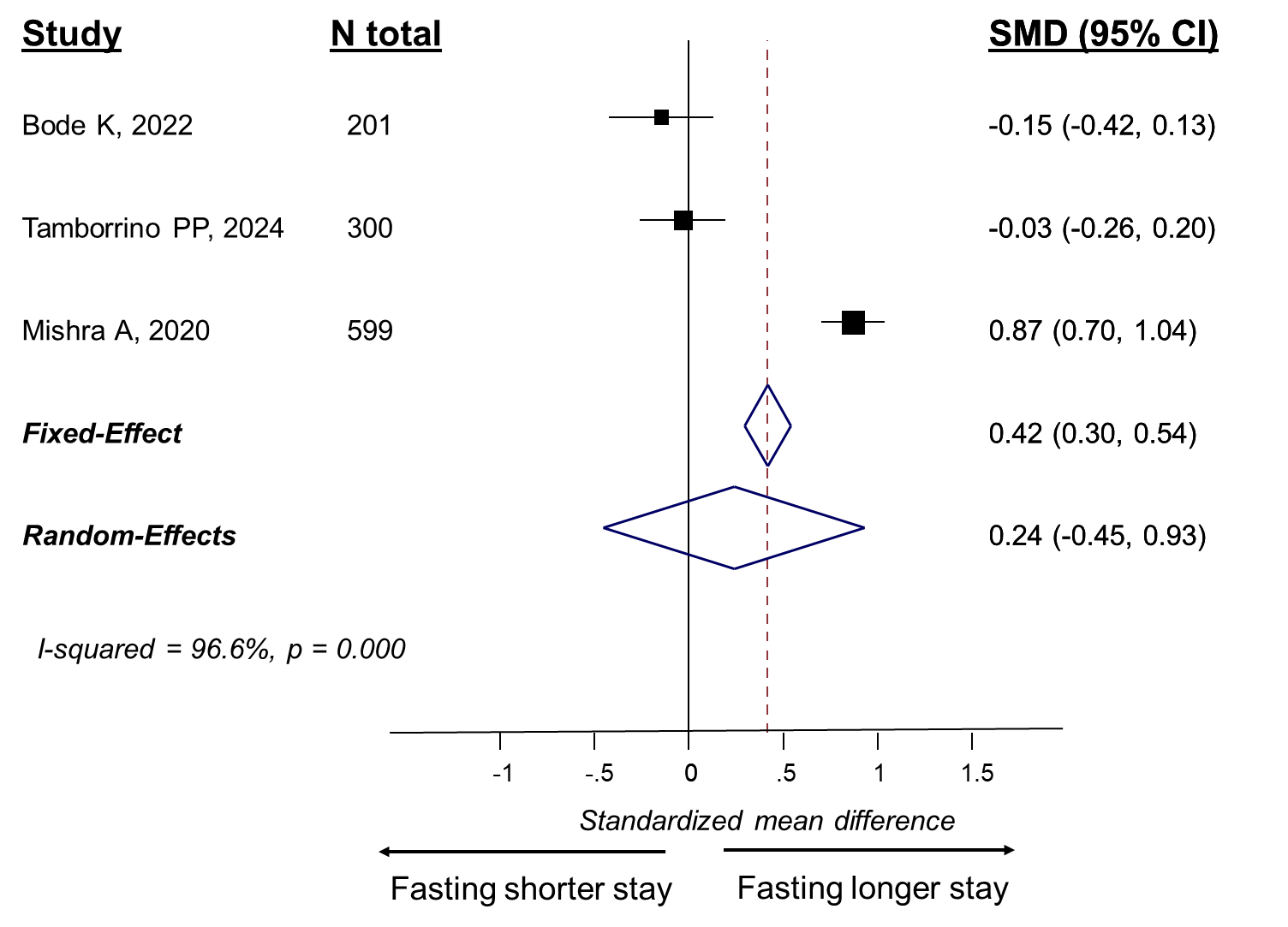


Supplement Figure 2. Seven clinical outcomes in 8 RCTs comparing fasting vs. non-fasting prior to cardiac procedures (pooled using fixed-effect meta-analysis model).


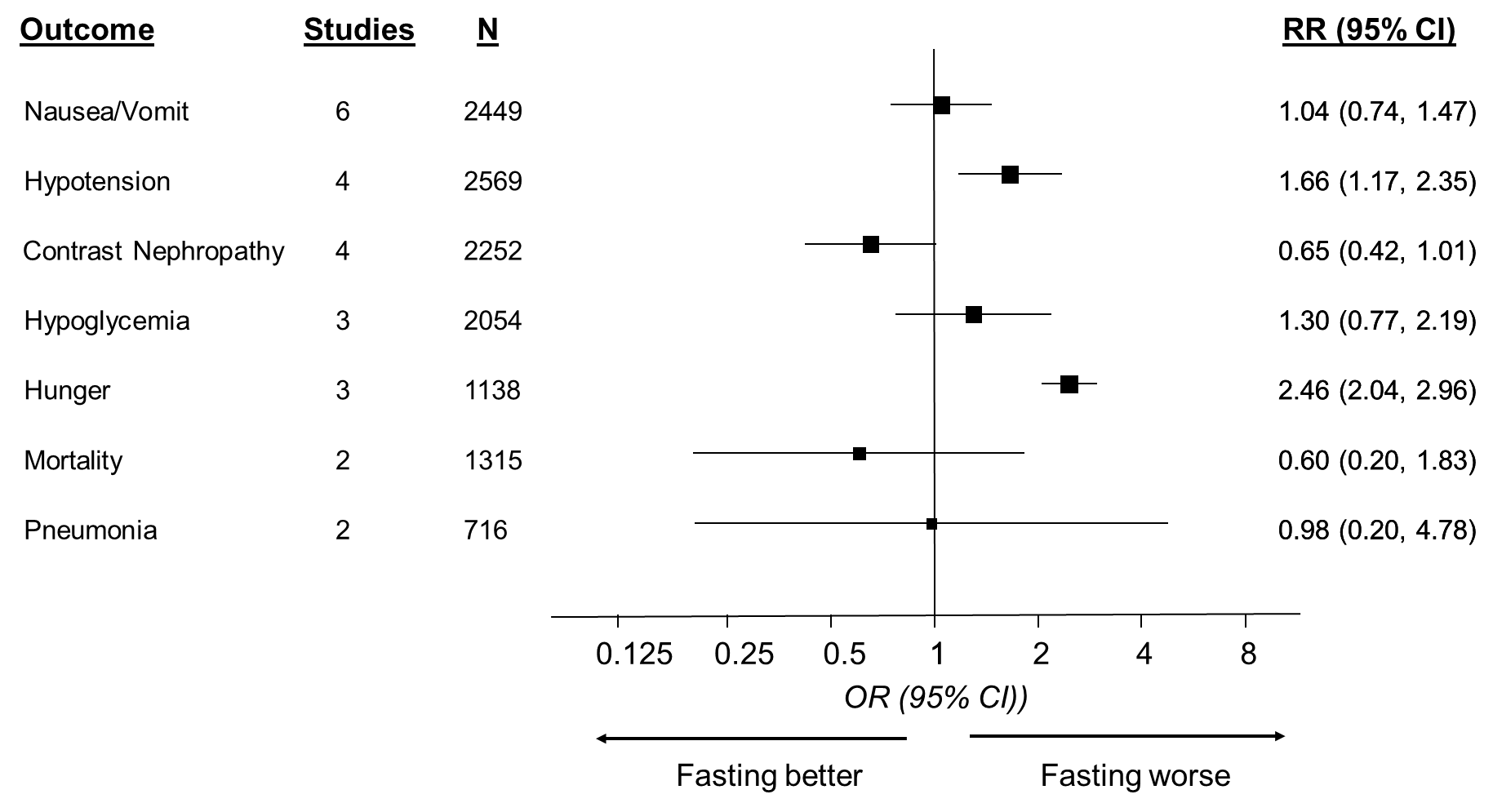


Supplement Figure 3. Funnel plot to assess publication bias across 6 studies included in meta-analysis of patient satisfaction score.


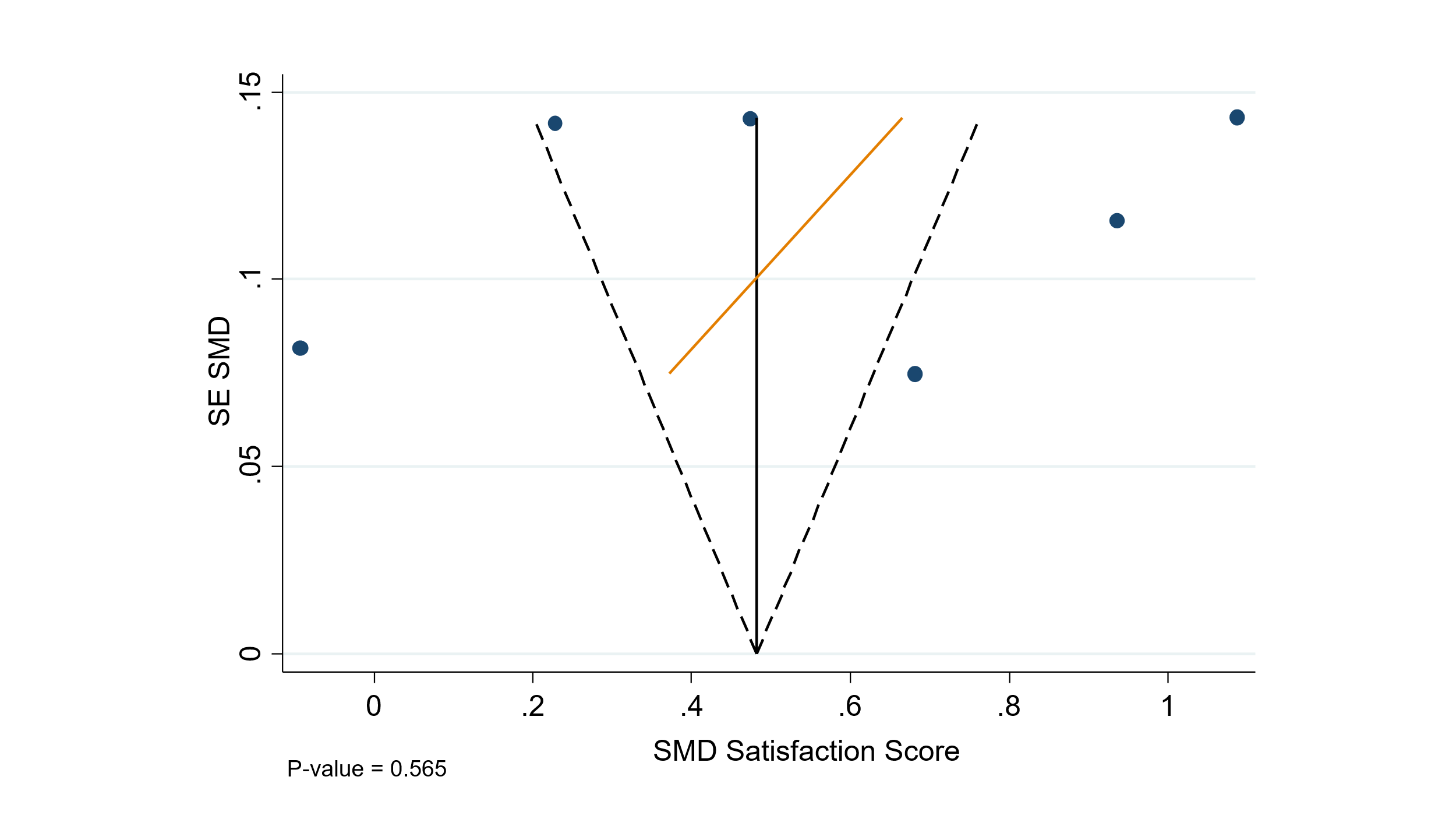


Supplement Figure 4. Influence analysis of 6 randomized trials included in meta-analysis of patient satisfaction score.


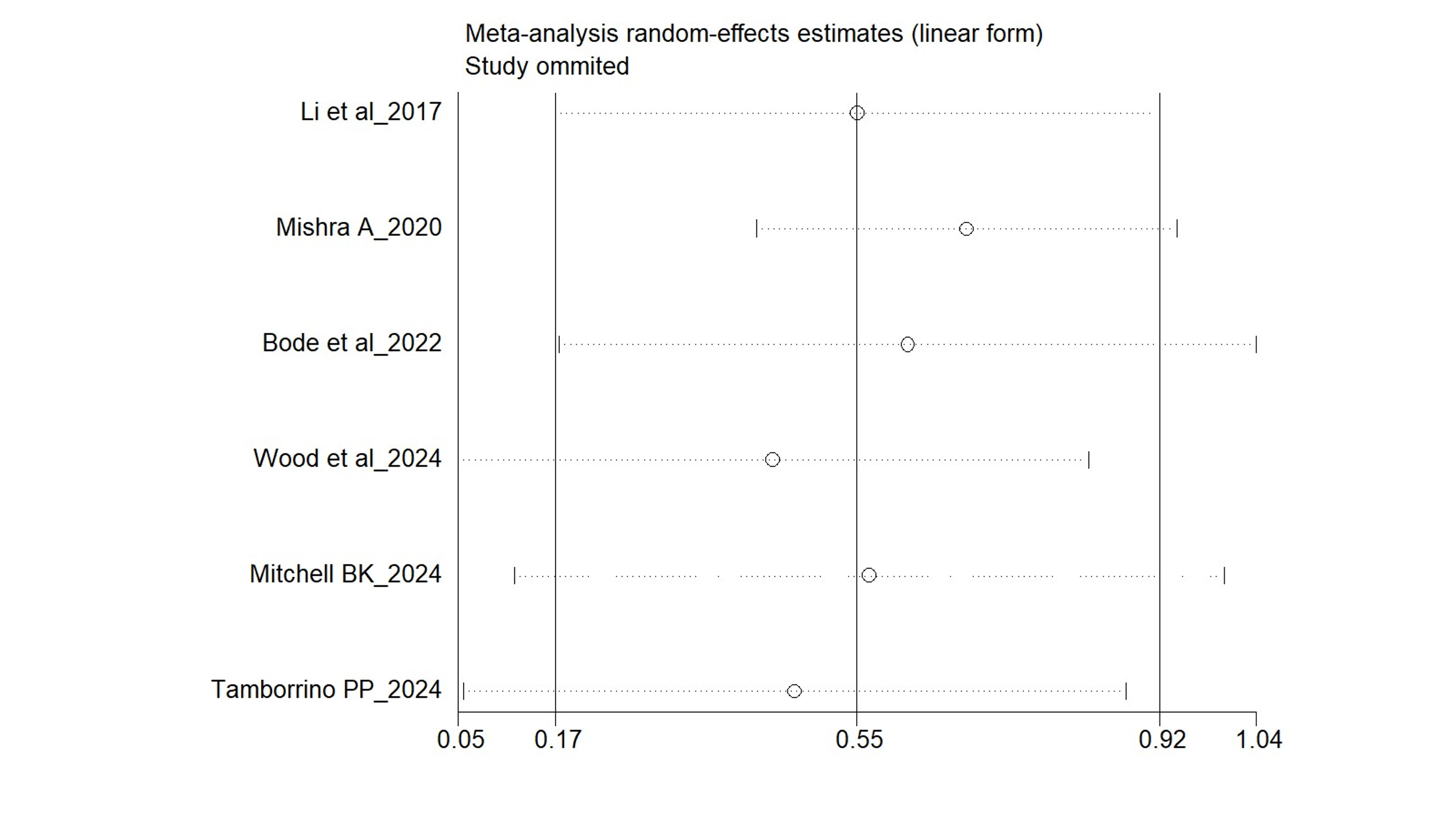


Supplement Figure 5. Sensitivity analysis for meta-analysis of patient satisfaction score, excluding two studies* that were reported as abstract only.


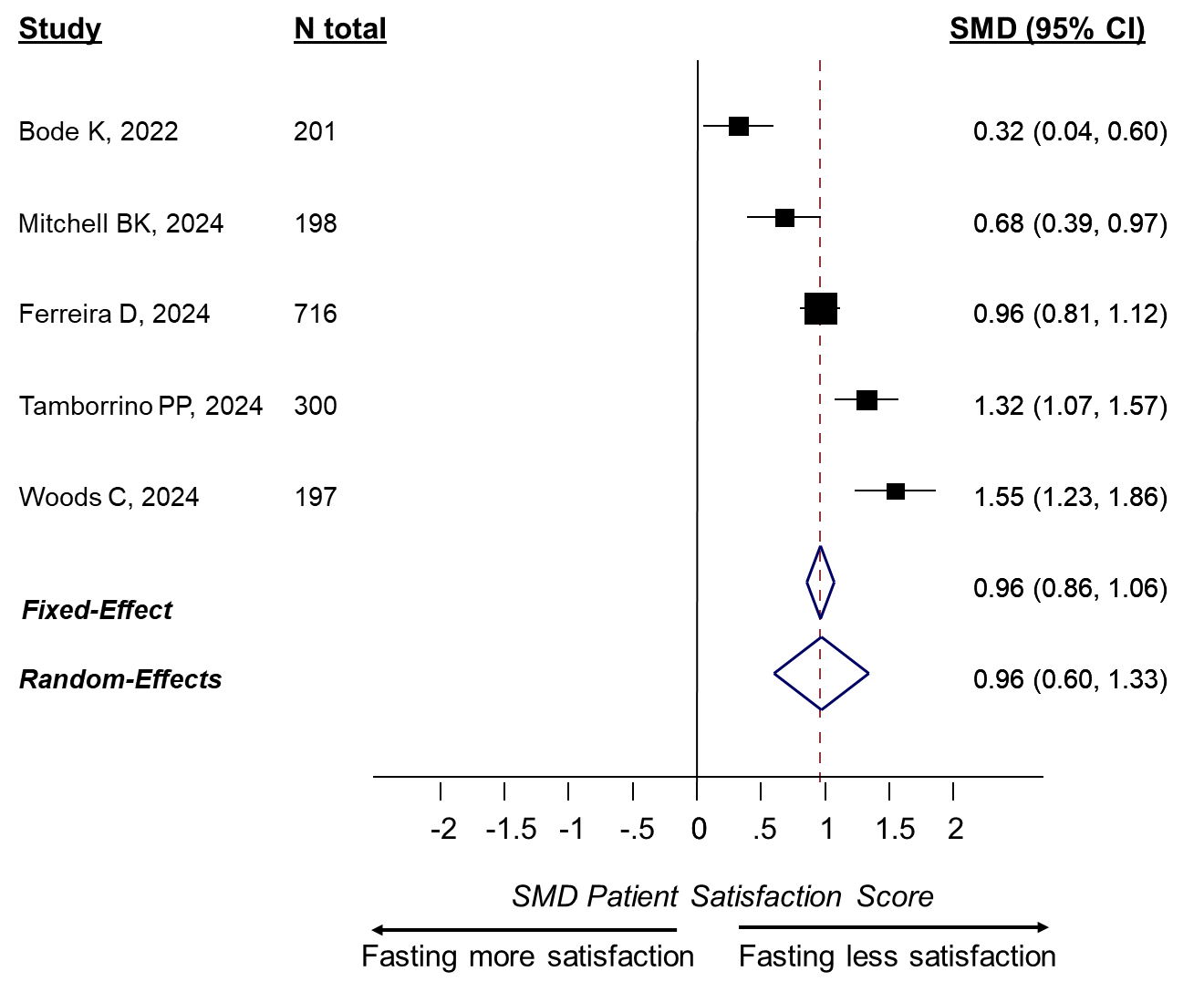


*A sensitivity analysis excluding Mishra A et al (2020) and Li Y et al (2017), which only available as abstracts of scientific conference presentations, yielded similar results.
